# Impact of Preoperative Anemia on Perioperative Outcomes in Patients Undergoing Elective Colorectal Surgery

**DOI:** 10.1155/2018/2417028

**Published:** 2018-05-08

**Authors:** Liu Liu, Lin Liu, Li-Chuang Liang, Zhi-qiang Zhu, Xiao Wan, Heng-bing Dai, Qiang Huang

**Affiliations:** ^1^Department of General Surgery, The First Affiliated Hospital of University of Science and Technology of China, Hefei, Anhui Province 230001, China; ^2^Department of Anesthesiology, The First Affiliated Hospital of University of Science and Technology of China, Hefei, Anhui Province, China; ^3^Department of General Surgery, Anhui Provincial Hospital Affiliated to the Anhui Medical University, Hefei, Anhui Province 230001, China

## Abstract

**Aim:**

To evaluate the impact of preoperative anemia (POA) on perioperative outcomes in patients undergoing elective surgery for colorectal cancer (CRC).

**Methods:**

A total of 326 CRC patients were enrolled. POA was defined as a hemoglobin (Hb) concentration ≤ 12 g/dl. Univariable and multivariable analyses were performed to assess the impact of POA on the risks of postoperative complications like surgical site infection (SSI).

**Results:**

Patients with colon cancer presented higher rate of POA than patients with rectal cancer (60% versus 40% for colon cancer versus rectal cancer). In addition, female patients and patients with large tumor mass (>4 cm) had a higher rate of POA than male patients and patients with small tumor (≤4 cm), respectively. Upon univariable analysis, CRC patients with POA had a higher rate of incisional SSI than patients without POA (12% versus 6%, *P* = 0.04). However, POA was not associated with other postoperative complications, like anastomotic leak, organ space SSI, and bleeding. Upon multivariable analysis, POA and stoma formation were identified as two independent risk factors for incisional SSI (OR (95%CI): 2.44 (1.09–5.49) for POA versus no POA and 2.64 (1.20–5.81) for stoma formation versus no stoma formation).

**Conclusions:**

POA was an independent risk factor for incisional surgical site infection after colorectal resection for CRC, and correcting POA should be considered before elective surgery.

## 1. Introduction

Surgery for colorectal cancer (CRC) has a high risk of morbidity (20%–40%) and mortality (1%–2%) [1, 2], which is mainly a result of postoperative infection. Postoperative infection included surgical site infection (SSI) and pulmonary and urinary tract infections. According to published reports, occurrence of SSI is as high as 25% in patients undergoing colorectal resection [3, 4]. Because SSI results in prolonged hospitalization and high medical cost [3], preventing and reducing the incidence of SSI are still important clinical issues.

Many host- and disease-related factors have been studied, and some of them are supposed to be predictive for occurrence of SSI. Obesity is a commonly reported risk factor for the development of SSI [5]. In addition, mechanical bowel preparation with oral antibiotics has also been suggested to reduce SSI and anastomotic leak [6]. Other risk factors for SSI include surgical type (laparoscopy versus open), intraoperative hypotension, and so on [3, 5].

Preoperative anemia (POA) is very common in CRC patients. It has been reported that the prevalence of POA is as high as 23.23% in Chinese CRC patients [7]. Additionally, a recent meta-analysis reported that about 39.1% of patients had POA [8]. POA has been suggested to be associated with increased occurrence of postoperative complications and mortality. Evidence from a meta-analysis reported that POA was associated with increased 2.9-fold risk of mortality, 3.75-fold risk of acute kidney injury, and 1.93-fold risk of infection after surgery [8]. Interestingly, a study by Jung et al. reported that the lowest postoperative hemoglobin (Hb) level, rather than POA, was the risk factor for postoperative complications in gastric surgery [9]. Whether POA is a risk factor for postoperative complications in elective colorectal resection remains uncertain. In this study, we retrospectively analyzed the impact of POA on risk of postoperative complications, especially SSI, based on the data of CRC patients undergoing elective surgery.

## 2. Patients and Methods

### 2.1. Patient Involvement

We retrospectively collected the clinical data of 405 patients who underwent elective colorectal surgery for pathologically confirmed colorectal cancer from January 2011 to December 2013 at the department of general surgery, the First Hospital Affiliated to the University of Science and Technology of China in China. This study is approved by the Ethical Committee of Anhui Provincial Hospital.

Eligible criteria for patient selection included pathologically conformed colorectal cancer and undergoing electively palliative or curative colorectal resection. Exclusion criteria were receiving preoperative chemoradiotheray (*n* = 23), distant metastasis (*n* = 16), a history of malignancy (*n* = 8), the situation like complete colorectal obstruction and perforation that required emergency surgery (*n* = 9), and receiving preoperative RBC transfusion in one month before admission (*n* = 7). Patients with systemic inflammatory response syndrome (*n* = 12) and blood systemic diseases (*n* = 4) were also excluded. Finally, 326 patients who underwent elective colorectal resection for CRC were enrolled.

### 2.2. Preoperative Treatment and Surgical Approach

All patients received prophylactic antibiotics according to the guideline for clinical application of antimicrobial agents in China [10]. Patients received intravenous prophylactic antibiotics 30 minutes before surgery and stopped antibiotic treatment in 48 hours after surgery. If there were clinical signs like fever, incision swelling and pain that indicated postoperative infection, bacteria culture was performed and an expert of infectious diseases in our hospital was invited to decide the treatment strategy of anti-infection. Mechanical bowel preparation was routinely administrated. Six surgeons performed surgery for these patients; all of the surgeons conducted more than 60 cases of colorectal surgery annually. All patients received electively curative or palliative colorectal resection. Curative resection was defined as R0 resection with lymph node dissection for colon cancer and as total mesorectal excision (TME) for rectal cancer. Palliative resection was conducted in patients with advanced CRC that invaded into adjacent tissues or organs like abdominal wall or bladder. Surgical approaches including laparoscopy-assisted, hand-assisted laparoscopy, and traditional open surgery were performed at the discretion of individual surgeon. Creation of a protective defunctioning stoma was based on the individual surgeon's usual practice. Wound protection was routinely conducted in all surgical procedures.

### 2.3. Data Collection and Variable Studied

We collected the following information from medical records: patients' age, gender, BMI, preoperative Hb, location of cancer (colon or rectum), American Society of Anesthesiologist (ASA) classification, preoperative comorbidity, abdominal surgical history, operative time, estimated blood loss, RBC (red blood cells) transfusion, surgical types (laparoscopy or open surgery), surgical procedure (colon or rectal resection), stoma formation, tumor diameter, and TNM stage. Patients were pathologically categorized by the 7th AJCC/TNM staging system (American Joint Committee on Cancer (AJCC)) [11].

POA was defined by a Hb level < 12 g/dl [12]. Patients who received RBC transfusion in one month before surgery were excluded from this study, because preoperative blood transfusion could distort the level of preoperative Hb. Overweight was defined as body mass index > 25 kg/m^2^ [13].

The primary endpoint was surgical site infection (SSI) rate within 30 days after surgery. SSI was defined by the Center for Disease Control and Definition ((CDC) http://www.anzjsurg.com/view/0/surgicalSiteInfectionCDCDef.html). SSI was categorized as being superficial, deep, and organ/space SSI.

Secondary endpoint included surgically related and systemic complications that occurred within 30 days after surgery. Surgically related complications included the complications directly caused by surgery, including bleeding, anastomotic leak, small bowel obstruction, ileus, and stoma stenosis. Systemic complications included pulmonary, cardiac, urinary tract, and neurological problems. There was no death for all patients.

### 2.4. Statistical Analysis

Data were shown as proportion or median (25% quartile–75% quartile). Continuous data was compared using the Mann–Whitney *U* test, and category data was compared by chi-square test or Fisher's exact test, as appropriate. Univariable analysis was conducted to analyze the impact of preoperative anemia on postoperative recovery and to assess factors influencing the occurrence of SSI. Finally, the variables that had a significant impact on the occurrence of SSI were used to build a multivariable logistic regression analysis. All statistical analyses were conducted using SPSS® version 22.0 software (SPSS, IBM). All statistical tests were two sided with *P* < 0.05 suggesting statistical significance.

## 3. Results

### 3.1. Characteristics of Patients

Among 326 patients with pathologically confirmed CRC, 154 patients (47.2%) were diagnosed with POA. The characteristics of included patients were shown in Table 1. Female patients and patients with colon cancer had a higher rate of POA. Median level of Hb was 10.4 g/dl in POA patients and 13.4 g/dl in non-POA patients (*P* < 0.01). In addition, POA patients had higher rate of hypertension than non-POA patients (*P* < 0.01). There was no statistical significance of age, BMI, ASA classification, and diabetes between the two groups of patients.

POA patients received more RBC transfusion than those without POA (25.9% versus 1.7%, *P* < 0.01) (Table 2). In addition, higher proportion of non-POA patients underwent laparoscopic surgery, while more proportion of POA patients received traditional open surgery. Patients with POA had larger tumor mass (>4 cm), while tumor mass in non-POA patients was smaller (69% versus 56% of patients with tumor mass > 4 cm, *P* = 0.02). Furthermore, more lymph nodes were harvested in non-POA patients than in POA patients (median: 9 versus 7, *P* < 0.01). There was no statistical significance of positive metastatic lymph node and TNM stage between the two groups of patients.

### 3.2. Perioperative Outcomes

Compared to patients without POA, patients with POA had a higher risk of incisional SSI (12% versus 6%, *P* = 0.04) (Table 3). Interestingly, there was no statistical significance of organ space SSI between two groups. Furthermore, POA did not increase the risks of anastomotic leak, postoperative bleeding, ileus and reoperation. Meanwhile, POA also did not increase the occurrence of systemic complications like pulmonary, cardiac and urinary tract complications. The length of postoperative hospitalization was also comparable between POA and non-POA patients (median: 8 versus 9 days for POA versus non-POA patients, *P* = 0.14).

### 3.3. Risk Factors Related to Incisional SSI

Upon univariable analysis, POA and stoma formation were significantly associated with increased risk of incisional SSI (*P* = 0.04 for POA and *P* < 0.01 for stoma formation, Table 4). Upon multivariable analysis (Table 5), POA and stoma formation increased 2.44-fold and 2.64-fold risk of incisional SSI, respectively (OR (95%CI): 2.44 (1.09–5.49) for POA and 2.64 (1.20–5.81) for stoma formation).

## 4. Discussion

High prevalence of anemia has been reported in surgical patients, and POA has been proved to increase occurrences of postoperative mortality and complications in cardiac, gastric and esophageal surgery [8]. In addition, POA also contributes to increased risk of postoperative infection in cardiac and orthopaedic surgery [14, 15]. In present study, we retrospectively investigated the impact of POA on the occurrence of postoperative complications in patients undergoing elective colorectal resection for CRC. The evidence from this study suggested that patients with POA had significantly higher risk of incisional SSI than non-POA patients. Taken into account confounding factors adjusted by multivariable analysis, preoperative anemia and stoma formation were identified as two independent factors for postoperative incisional SSI.

The prevalence of POA greatly varies in CRC patients due to different locations of cancer lesion. Overall rate of CRC-related anemia ranges from 20% to 50% [16]. In a study by Dunne et al., POA was analyzed in 311 CRC patients, and rates of POA was 47% in right-sided colon cancer, 34% in left-sided colon cancer and 19% in rectal cancer. In addition, patients with right-sided colon cancer had significantly advanced pathological stage when comparing to patients with left-sided colonic and rectal cancer [17]. In accordance with published studies [8, 17], we observed that more POA patients were with colon cancer (60%), while more non-POA patients were with rectal cancer (78%).

Multiple factors contribute to the incidence of cancer-related anemia in CRC patients. Chronic blood loss from tumor mass is the important reason for cancer-related anemia, because cancer patients with anemia always show significantly higher ferritin and hepcidin levels than general peoples, which suggests iron deficiency in these patients [16]. However, iron supplementation cannot effectively correct anemia [18], which suggests that cancer-related anemia is as a result of complex effects of multiple factors, like age, gender and cancer stage. A study by Gao et al. analyzed 918 patients with solid cancers, and results indicated that males, older patients, less diet and advanced stage of cancers significantly increased the incidence of POA in Chinese patients [7]. A recent study analyzed 201 CRC patients, and results of this study suggested that POA patients had larger size of tumor mass and more blood transfusion than patients without POA [19]. In our study, female patients and patients with colon cancer were at high risk for incidence of preoperative anemia. In addition, in consistent to published study [19], tumor mass was significantly larger in POA patients than non-POA patients.

High prevalence of POA causes great attention of surgeons, and many clinical studies have been conducted to investigate its impact on surgical recovery [8, 14, 20]. Baron et al. analyzed 39,309 non-cardiac surgery patients, and they found that POA significantly increased risk of postoperative mortality, and resulted in prolonged hospitalization [20]. Similarly, another study analyzed 36,658 patients undergoing coronary artery bypass graft surgery, and results of this study suggested that POA patients had as 2.37-fold and 1.87-fold risks of postoperative mortality and morbidity as the patients POA [14]. A meta-analysis suggested that POA contributed to 2.9-fold risk of postoperative mortality and 1.93-fold risk of infection when comparing to non-POA patients [8]. In present study, CRC patients with POA were 2.44-fold risk of incisional SSI than non-POA. Interestingly, we did not find significant associations between POA and organ space SSI and anastomtic leak. Long-term impact of POA on prognosis of CRC needs further investigation.

Although increasing evidence suggested that POA was associated with poor outcomes after surgery [8, 12, 20], the mechanisms remain unclear. Experimental studies have confirmed that anemia induced decreased oxygen delivery, elicited multiple organ hypoxia, like the brain and kidney, and finally caused organ dysfunction [21, 22]. Consistent to the findings of experimental studies, clinical trials have proved that anemia caused acute kidney injury and increased postoperative infection, which were closely associated with anemia-induced hypoxia [8]. Similarly, increased risk of incisional SSI was observed in POA patients in the present study. Besides, POA increased risk of allogeneic blood transfusion which suppresses cellular immunity and causes SSI. Janssen et al. analyzed 3721 patients who underwent laminectomy and/or arthrodesis of the lumbar spine. This study showed that blood transfusion increased 3-fold risk of SSI, 4.9-fold risk of urinary tract infection, and 6.5-fold pneumonia [23]. In contrast, patients undergoing blood transfusion were not statistically different between incisional SSI and nonincisional SSI groups in our study.

The present study has some limitations. First, this study is retrospective and encounters some inherent bias. In addition, this study could not determine the causality of POA in incisional SSI for CRC patients. Therefore, prospective studies are necessary in the future. Second, the effects of some unknown or unmeasured confounders on the association between POA and incisional SSI could not be ruled out. However, owing to the robust results of this study, it seems that confounding factors that were not included in analysis were unlike to change the results.

In summary, this study suggested that POA (Hb < 12 g/dL) was associated with an increased risk of incisional SSI after elective CRC surgery, and correcting preoperative anemia before surgery should be considered. Prospective high-quality studies were needed to confirm our findings in the future.

## Figures and Tables

**Table 1:** Preoperative variables.

Clinical features	Total (*n*, %)	Anemia group (*n*, %)	Nonanemia group (*n*, %)	*P* value
Patients	326	154	172	
Age (years)				0.20
≤65 years	197 (60%)	87 (57%)	110 (64%)	
>65 years	129 (40%)	67 (43%)	62 (36%)	
Gender (male/female)				<0.01
Males	191 (59%)	61 (40%)	130 (73%)	
Females	135 (41%)	93 (60%)	42 (27%)	
BMI (kg/m^2^)				0.14
<25	285 (87%)	139 (90%)	146 (85)	
≥25	41 (13%)	15 (10%)	26 (15%)	
RBC (×10^12^/L)^†^	4.2 (3.8–4.6)	3.8 (3.5–4.0)	4.5 (4.3–4.7)	<0.01
Hemoglobin (HB, g/dl)	12.1 (10.4–13.5)	10.4 (9.1–11.2)	13.4 (12.7–14.3)	<0.01
Location of cancer				<0.01
Colon	141 (43%)	93 (60%)	38 (22%)	
Rectum	195 (57%)	61 (40%)	134 (78%)	
ASA classification				0.13
ASA-1/2	309 (6%)	149 (97%)	160 (93%)	
ASA-3	17 (89%)	5 (3%)	12 (7%)	
Comorbidities				
Hypertension	75 (23%)	48 (31%)	27 (16%)	<0.01
Diabetes	30 (9%)	16 (10%)	13 (7%)	0.47
Cardiac diseases	14 (4%)	9 (6%)	5 (3%)	0.19
Respiratory diseases	12 (4%)	4 (3%)	8 (5%)	0.33
Renal diseases	2 (0.6%)	1 (0.7%)	0 (0%)	0.47
Cerebral diseases	7 (2%)	4 (3%)	3 (2%)	0.71
Abdominal surgical history	54 (17%)	25 (16%)	29 (17%)	0.52

^†^RBC: red blood cells.

**Table 2:** Intraoperative variables.

	Anemia group (*n* = 154)	Nonanemia group (*n* = 172)	*P* value
Operative time	140 (120–180)	138 (115–180)	0.90
Estimated blood loss	100 (80–150)	100 (50–150)	0.41
Perioperative RBC transfusion, *n* (%)	40 (25.9%)	3 (1.7%)	<0.01
Units of RBC transfusion	3 (2–6)	4 (2–4)	<0.01
Types of operation, *n* (%)			<0.01
Laparoscopic surgery^†^	60 (39%)	109 (63%)	
Open surgery	94 (61%)	63 (37%)	
Surgical procedure, *n* (%)			<0.01
Colon resection	93 (60%)	38 (22%)	
Rectum resection	61 (40%)	135 (78%)	
Stoma formation, *n* (%)	36 (23%)	51 (30%)	0.20
Maximum diameter of cancer (cm)			0.02
<4 cm	47 (31%)	75 (44%)	
≥4 cm	107 (69%)	97 (56%)	
T stage, *n* (%)			0.25
T1	4 (3%)	6 (4%)	
T2	19 (12%)	35 (20%)	
T3	98 (64%)	98 (57%)	
T4	33 (21%)	33 (19%)	
Number of nodal harvest	9 (6–12)	7 (4–11)	<0.01
Patients with nodal metastasis, *n* (%)	52 (34%)	70 (41%)	0.31
TNM stage, *n* (%)			0.58
I/II	96 (62%)	102 (59%)	
III/IV	58 (38%)	70 (41%)	

^†^Included laparoscopy-assisted and hand-assisted laparoscopic colorectal resection for colorectal cancer (CRC).

**Table 3:** Postoperative complications between POA and non-POA patients.

	POA group (*n* = 154)	Non-POA group (*n* = 172)	*P* value
*Surgically related complications*			
SSI^†^	19 (12%)	13 (8%)	0.15
Incisional SSI	19 (12%)	10 (6%)	0.04
Organ space SSI	4 (3%)	6 (3%)	0.75
Ileus	3 (2%)	6 (3%)	0.51
Small bowel obstruction	1 (1%)	0 (0%)	0.47
Anastomotic leakage	3 (2%)	5 (3%)	0.73
Bleeding	2 (1%)	3 (2%)	1.00
Stoma stenosis	2 (1%)	4 (2%)	0.69
*Systemic complications*			
Pulmonary problem	4 (3%)	2 (1%)	0.43
Cardiac problem	1 (1%)	0 (0%)	0.47
Urinary problem	1 (1%)	3 (2%)	0.63
Neurologic problem	0 (0%)	0 (0%)	NA
Others	2 (1%)	1 (1%)	0.60
Reoperation	3 (2%)	7 (4%)	0.35
Postoperative death	0 (0%)	0 (0%)	NA
Number of complications^¶^	47 (31%)	32 (19%)	
1	20 (13%)	26 (15%)	0.58
2	3 (2%)	4 (2%)	1.00
3 or more	3 (2%)	2 (1%)	0.67
Infectious complications	23 (15%)	20 (12%)	0.39
Postoperative hospitalization	8 (7–10)	9 (8–11)	0.14

^†^SSI: surgical site infection; ^¶^Number of complications, per patient with the number of complications after surgery; POA: preoperative anemia; NA: not available.

**Table 4:** Univariable analysis of factors linked to incisional SSI^†^.

		Incisional SSI (*n*, %)	No incisional SSI (*n*, %)	*P* value
Patients (*n*)		29	297	
POA	Yes	19 (66%)	135 (46%)	0.04
	No	10 (34%)	162 (54%)	
RBC transfusion	Yes	3 (10%)	33 (11%)	0.90
	No	26 (90%)	264 (89%)	
Age	≤65 years	16 (55%)	181 (61%)	0.54
	>65 years	13 (45%)	116 (39%)	
Gender	Males	18 (62%)	173 (58%)	0.69
	Females	11 (38%)	124 (42%)	
BMI	<25 kg/m^2^	23 (79%)	262 (88%)	0.17
	≥25 kg/m^2^	6 (21%)	35 (12%)	
Location of cancer	Colon	9 (31%)	122 (41%)	0.29
	Rectum	20 (69%)	175 (59%)	
ASA classification	ASA-1/2	27 (93%)	282 (95%)	0.67
	ASA-3	2 (7%)	15 (5%)	
Hypertension	Yes	8 (28%)	67 (23%)	0.54
	No	21 (72%)	230 (77%)	
Diabetes	Yes	2 (7%)	28 (9%)	0.65
	No	27 (93%)	269 (91%)	
Cardiac diseases	Yes	2 (7%)	13 (4%)	0.54
	No	28 (93%)	284 (96%)	
Respiratory diseases	Yes	0 (0%)	12 (4%)	0.27
	No	29 (100%)	285 (96%)	
Renal diseases	Yes	1 (4%)	0 (0%)	0.09
	No	28 (96%)	296 (100%)	
Cerebral diseases	Yes	0 (0%)	7 (2%)	1.00
	No	29 (100%)	290 (98%)	
Abdominal surgical history	Yes	7 (24%)	47 (16%)	0.25
	No	22 (76%)	250 (84%)	
Types of operation	Laparoscopic surgery	15 (52%)	154 (36%)	0.75
	Open surgery	14 (48%)	163 (64%)	
Stoma formation	Yes	12 (41%)	24 (8%)	<0.01
	No	17 (59%)	273 (92%)	
Surgical resection	Colon resection	8 (28%)	123 (41%)	0.15
	Rectum resection	21 (72%)	174 (59%)	
Cancer size(cm)	<4	9 (31%)	113 (38%)	0.46
	≥4	20 (69%)	184 (62%)	
T stage	T1/T2	7 (24%)	57 (19%)	0.52
	T3/T4	22 (76%)	240 (81%)	
AJCC-TNM stage	I/II	22 (76%)	176 (59%)	0.08
	III/IV	7 (24%)	121 (41%)	

^†^SSI: surgical site infection.

**Table 5:** Multivariate analysis of risk factors linked to incisional SSI.

	*P* value	OR	95%CI
Anemia (yes versus no)	0.03	2.44	1.09–5.49
Stoma formation (yes versus no)	0.02	2.64	1.20–5.81
